# Reviewer acknowledgement 2013

**DOI:** 10.1186/2045-7022-4-5

**Published:** 2014-02-05

**Authors:** Clive Grattan

**Affiliations:** 1 Norfolk and Norwich University Hospital, Norwich, UK

## Abstract

**Contributing reviewers:**

The editors of *Clinical and Translational Allergy* would like to thank all of our reviewers who have contributed to the journal in Volume 3 (2013).

## 

Ioana Agache

Romania

Isabella Annesi-Maesano

France

Riccardo Asero

Italy

Claus Bachert

Belgium

Iaria Baiardini

Italy

Isil Barlan

Turkey

Sevim Bavbek

Turkey

Roberto Berni Canani

Italy

M. Beatrice Bilò

Italy

Apostolos Bossios

Sweden

Jean Bousquet

France

Fulvio Braido

Italy

Randolf Brehler

Germany

Victoria Cardona

Spain

Jan Ceuppens

Belgium

Mirna Chehade

United States of America

Alfonso Del Cuvillo Bernal

Spain

Audrey Dunn Galvin

Ireland

Peter Eng

Switzerland

Mercedes Escarrer Jaume

Spain

Luis Escribano

Spain

Francisco Feo-Brito

Spain

Marta Ferrer

Spain

Penny Fitzharris

New Zealand

Hiroyuki Fujita

Japan

Roy Gerth van Wijk

Netherlands

John Gibson

United Kingdom

Mar Guilarte

Spain

Laurie Harada

Canada

Dolores Hernandez

Spain

Manuel Hernández-González

Spain

Hal Hoffman

United States of America

Karin Hoffmann-Sommergruber

Austria

Kathryn Hulse

United States of America

Maria Dolores Ibáñez Sandín

Spain

Lars Jacobsen

Denmark

Maria Jenmalm

Sweden

Omer Kalayci

Turkey

Jörg Kleine-Tebbe

Germany

Greg Ladics

United States of America

Jing Li

China

Allan Linneberg

Denmark

Ramon Lleonart

Spain

David Luyt

United Kingdom

Joanna Makowska

Poland

Patrick Mallia

United Kingdom

Hans-Jørgen Malling

Denmark

Adriano Mari

Italy

Nadine Marrouche

United Kingdom

Marcus Maurer

Germany

Michael McDermott

United Kingdom

Louise Michaelis

United Kingdom

Ralph Mösges

Germany

Marek Niedoszytko

Poland

Antonio Nieto

Spain

Liam O'Mahony

Switzerland

Lars Poulsen

Denmark

Graham Roberts

United Kingdom

Albert Roger

Spain

Bert Ruiter

United States of America

Cansin Sackesen

Turkey

Anna Sala Cunill

Spain

Rohit Saluja

Germany

Glenis Scadding

United Kingdom

Knut Schäkel

Germany

Stephan Scheurer

Germany

Sven Seys

Belgium

Michael Shields

United Kingdom

Elisabeth Stahl

Sweden

Kazunari Sugita

Switzerland

Massimo Triggiani

Italy

Paul Turner

United Kingdom

Antonio Valero

Spain

Carina Venter

United Kingdom

De Yun Wang

Singapore

Marcin Wawrzyniak

Switzerland

Thomas Werfel

Germany

Jonathan White

United Kingdom

Harry Wichers

Netherlands

